# Construction and validation of a nomogram model for predicting 60-day mortality in patients with acute myeloid leukaemia

**DOI:** 10.3389/fonc.2026.1846499

**Published:** 2026-09-03

**Authors:** Man Yang, Youmei Zi, Xuyang Dai, Yongqiang Liu, Luyao Zhu, Lihua Wang, Yuan Zhang, Guoqing Lv, Sun Wu, Weihong Lu, Yan Huang

**Affiliations:** 1 The First Affiliated Hospital of Henan Medical University, Xinxiang, Henan, China; 2 Xinxiang Key Laboratory of Molecular Diagnosis and Treatment of Leukemia, Xinxiang, Henan, China; 3 Xinxiang Key Laboratory of Molecular Diagnosis and Treatment of Lymphoma, Xinxiang, Henan, China; 4 The Center of Hematopoietic Stem Cell Transplantation and Immunotherapy, The First Affiliated Hospital of Henan Medical University, Xinxiang, Henan, China

**Keywords:** 60-day early mortality, acute myeloid leukaemia, cox proportional hazards regression, European leukaemia network risk stratification, extramedullary disease, nomogram, Shapley additive explanations interpretable machine learning

## Abstract

**Objective:**

To construct and internally validate a nomogram for predicting 60-day all-cause mortality in adult patients with *de novo* acute myeloid leukaemia (AML) and to compare its performance with European Leukaemia Network (ELN) 2022 risk stratification.

**Methods:**

Clinical data from 140 patients with newly diagnosed *de novo* AML treated at The First Affiliated Hospital of Henan Medical University between 2020 and 2025 were retrospectively analysed. Patients were allocated by stratified random split into a training cohort (*n* = 98; 21 death events) and an internal validation cohort (*n* = 42; 8 death events). Univariate and multivariate Cox proportional hazards regression were used consistently for time-to-event analysis. Proportional hazards assumptions were assessed using Schoenfeld residuals. Collinearity diagnosis, age-stratified subgroup analysis, treatment-intensity stratification, 200 bootstrap iterations and stratified 10-fold cross-validation were performed to evaluate model stability and reduce optimism. Discrimination, calibration, clinical utility and interpretability were assessed using time-dependent area under the curve (AUC), C-index, calibration curves, decision curve analysis and Shapley Additive Explanations (SHAP) analysis.

**Results:**

Twenty-nine patients (20.7%) died within 60 days. The final multivariate Cox model identified extramedullary disease (EMD) (hazard ratio [HR]=8.24, 95% confidence interval [CI]:1.91–35.63, P = 0.005), fibrinogen (per 100 mg/dL: HR = 1.12, 95%CI:1.04–1.21, P = 0.002), ferritin (per 200 ng/mL: HR = 1.09, 95%CI:1.02–1.17, P = 0.012) and number of positive prognostic genes (HR = 0.59, 95%CI:0.41–0.86, P = 0.006) as independent predictors. Bootstrap-corrected AUC/C-index values were 0.88/0.79 in the training cohort and 0.84/0.76 in the validation cohort; stratified 10-fold cross-validation yielded an average AUC of 0.87. The nomogram outperformed ELN 2022 risk stratification alone (AUC: 0.95 vs 0.74, P<0.001); SHAP analysis ranked EMD as the dominant feature.

**Conclusion:**

This four-variable Cox nomogram showed favourable internally validated discrimination, calibration, clinical net benefit and interpretability for individualised 60-day mortality prediction in adult *de novo* non–acute promyelocytic leukaemia AML. Multicentre prospective external validation is warranted.

## 1 Introduction

Acute myeloid leukaemia (AML) is a highly heterogeneous haematologic malignancy characterised by clonal expansion of immature myeloid blasts in bone marrow and peripheral blood (1). The malignancy carries a prominent early mortality burden, concentrated within 4–8 weeks post-diagnosis prior to completion of induction chemotherapy. Despite progressive advances in hypomethylating agents, targeted molecular therapy and allogeneic haematopoietic stem cell transplantation, early death (ED) rates remain unacceptably high, reaching 15%–30% among older and high-risk AML subgroups (2–5). Timely and accurate stratification of patients at elevated ED risk is indispensable for designing individualised induction and supportive care regimens.

The European Leukaemia Network (ELN) 2017/2022 risk stratification currently represents the standard prognostic framework for AML, relying exclusively on cytogenetic and molecular aberrations to predict long-term relapse and overall survival (OS) (6, 7). However, this system was not originally designed to quantify short-term all-cause mortality during induction therapy. Cumulative evidence indicates that ELN classification alone lacks sufficient specificity to predict 60-day ED, necessitating the integration of acute clinical phenotypes, organ dysfunction markers and inflammatory and coagulation biomarkers to capture multidimensional ED risk drivers (8, 9). Consistent risk factors for AML-related ED identified across multicohort analyses include advanced age, poor Eastern Cooperative Oncology Group (ECOG) performance status, multiorgan impairment, high tumour burden, adverse ELN molecular risk and dysregulated coagulation.

Extramedullary disease (EMD), defined as leukemic blast infiltration outside bone marrow and peripheral blood, has emerged as a robust independent adverse prognostic phenotype. Large multicentre cohorts have confirmed that patients with EMD exhibit elevated white blood cell (WBC) counts and lactate dehydrogenase (LDH) levels, and frequent co-occurrence of *FLT3-ITD*, *NPM1* and *PTPN11* mutations. A recent meta-analysis validated EMD as a powerful negative prognostic marker, associated with shortened OS (hazard ratio [HR]=1.49) and event-free survival (EFS) (HR = 1.38), with isolated extramedullary infiltration conferring the worst clinical outcomes (HR = 1.88) (10, 11).

Coagulopathy and systemic hyperinflammation are core mechanistic drivers of AML-related ED. Disseminated intravascular coagulation (DIC), characterised by elevated fibrinogen (FIB) and D-dimer (DD), correlates with high leukemic burden and independently predicts haemorrhagic/thrombotic complications and 30–60-day mortality (12). Elevated FIB reflects systemic hyperinflammation and microvascular thrombosis, propagating multiorgan perfusion failure. Ferritin, an acute-phase reactant and iron overload biomarker, drives leukemic proliferation, immunosuppression and chemotherapy resistance via modulation of reactive oxygen species and ferroptosis signalling (13). Proteomic signatures such as the leukaemia inflammatory risk score outperform traditional single-biomarker models for early mortality prediction.

Beyond laboratory and clinical parameters, the AML molecular landscape – specifically the quantity and combination of favourable mutations – adds granular prognostic information. Next-generation sequencing (NGS) demonstrates that high-risk multigene co-mutations and elevated measurable residual disease variant allele frequency are associated with aggressive leukemic clones and increased relapse risk (14, 15).

Existing prediction tools rarely integrate extramedullary infiltration, coagulation/inflammatory biomarkers and favourable molecular mutation burden simultaneously to specifically quantify 60-day ED risk. Traditional ELN stratification ignores clinical inflammatory and organ-damage markers, whereas single-biomarker models fail to capture the multifactorial nature of early mortality. Nomograms convert complex multivariate regression outputs into intuitive, bedside-operable visual scoring systems, widely validated across oncology for prognostic prediction. However, conventional nomograms lack transparent internal decision logic, which limits clinicians’ trust. Shapley Additive Explanations (SHAP) interpretable machine learning quantifies individual feature contributions and global variable importance while maintaining predictive accuracy, thereby resolving the interpretability limitations of black-box statistical models (16, 17).

This study constructs and rigorously internally validates a Cox regression–based nomogram predicting 60-day all-cause mortality in patients with *de novo* AML. We comprehensively screen baseline clinical, laboratory, inflammatory, coagulation and NGS molecular variables to identify independent ED predictors. Multiple robust internal validation approaches, including bootstrap resampling and stratified 10-fold cross-validation, are used to mitigate overfitting bias due to limited sample size and endpoint events. We conduct a head-to-head performance comparison with the standard ELN 2022 risk stratification and use SHAP analysis to dissect the model’s decision-making pathways. A real clinical case demonstration is incorporated to enhance clinical operability, providing a quantitative, transparent and validated decision-support tool for early identification and intervention of patients with ultra-high risk for AML.

## 2 Materials and methods

### 2.1 Study design and ethical approval

This single-centre, retrospective observational cohort study enrolled consecutive patients with newly diagnosed AML admitted to the Department of Haematology, The First Affiliated Hospital of Henan Medical University, between January 2020 and December 2025. The research protocol received ethical approval from the Institutional Ethics Committee of The First Affiliated Hospital of Henan Medical University (approval number: EC-LW026-032). All research procedures complied with the Declaration of Helsinki. Given the retrospective study design and complete anonymisation of all patient clinical data, the institutional ethics committee waived the requirement for written informed consent.

### 2.2 Study population and selection criteria

The inclusion criteria included newly diagnosed primary *de novo* AML confirmed by bone marrow aspirate, biopsy and World Health Organization (WHO) 2016 haematologic malignancy classification criteria; complete baseline demographic, clinical, laboratory, imaging and NGS molecular testing data available at diagnosis; age ≥18 years; and complete follow-up data covering at least 60 days post-diagnosis, or confirmed all-cause death occurring within the 60-day follow-up window.

The exclusion criteria included acute promyelocytic leukaemia (APL, FAB M3 subtype); concurrent second primary malignant tumour or uncontrolled severe autoimmune disease; prior anti-leukaemia chemotherapy or targeted therapy (treatment-experienced patients, confounding baseline prognostic assessment); secondary AML transformed from myeloproliferative neoplasms, myelodysplastic syndrome or patients with prior radiotherapy/chemotherapy exposure: secondary AML exhibits distinct leukemic clone biology, inflammatory baseline and iron metabolism profiles compared with primary *de novo* AML, with divergent ED mechanisms (exclusion minimised cohort biological heterogeneity, as fully justified in the Discussion section); baseline key predictive variable missing rate exceeding 20%; and lost to follow-up, transferred to external hospitals or voluntarily withdrew follow-up before completion of the 60-day endpoint without recorded death events.

**Figure 1:**
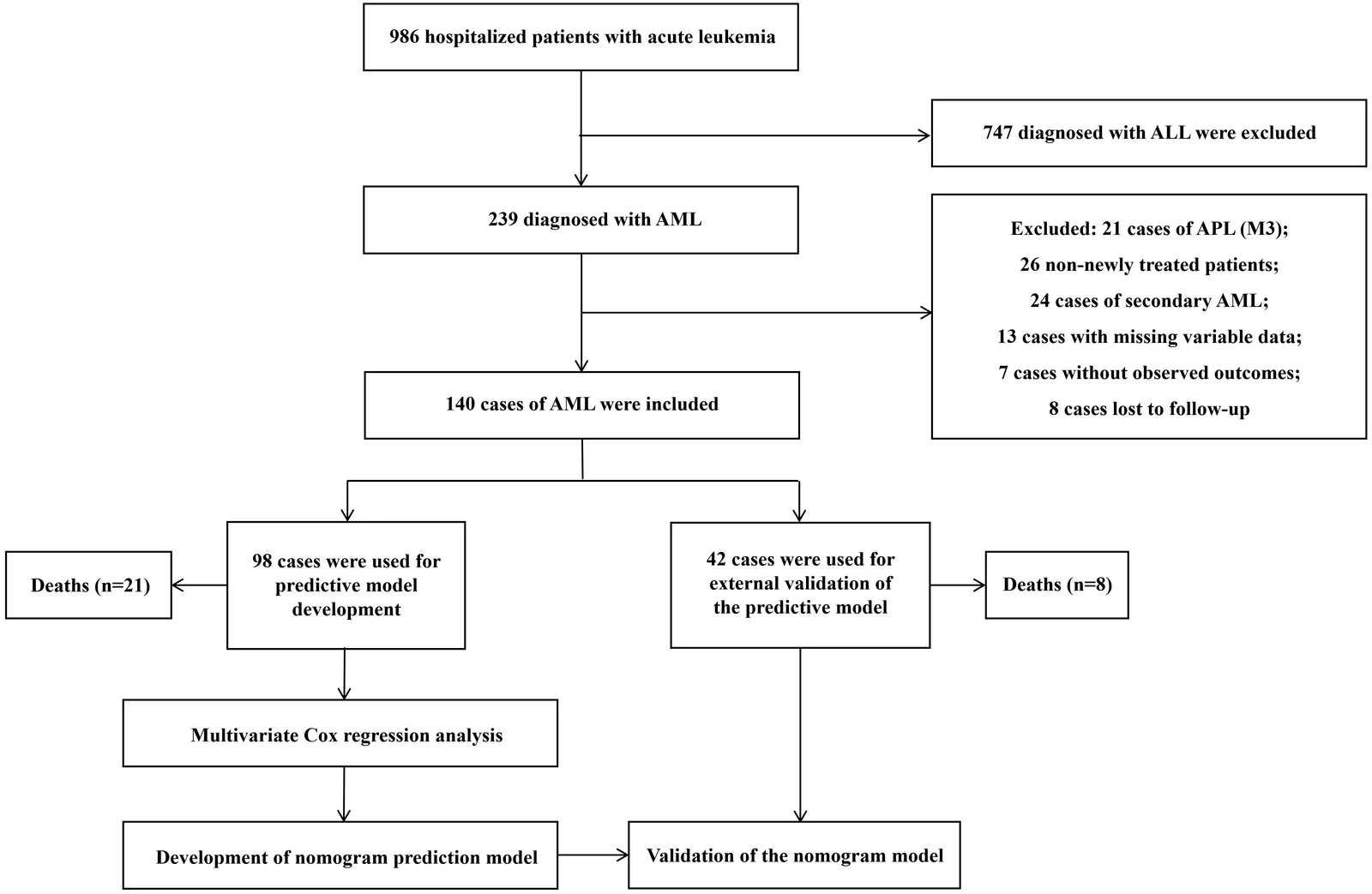
Patient screening and stratified cohort allocation flowchart for adult patients with *de novo* non–acute promyelocytic acute myeloid leukaemia.

Initial screening identified 986 hospitalised patients with acute leukaemia. Seven hundred forty-seven patients with acute lymphoblastic leukaemia were excluded, retaining 239 patients with AML. Further screening excluded 99 patients: 21 with APL/M3, 26 with treatment-experienced AML, 24 with secondary AML, 13 with >20% of core baseline variables missing, 7 with incomplete 60-day follow-up and 8 lost to follow-up. A total of 140 patients with primary *de novo* AML were ultimately enrolled. The full cohort underwent a stratified random split (stratified by a binary 60-day mortality endpoint) at a 7:3 ratio via a random number generator: training set (*n* = 98) (twenty-one 60-day death events) and internal hold-out validation set (*n* = 42) (eight 60-day death events). A complete research flowchart (**Figure 1**) visualises the patient screening and cohort grouping procedures.

### 2.3 Data collection and standardised variable definition

All baseline data were extracted from hospital electronic medical records at the time of AML diagnosis, before induction chemotherapy.

#### 2.3.1 Demographic, clinical and laboratory variables

Baseline demographic and laboratory data are summarised in **Table 1**.Demographic data included age, biological sex, body mass index, smoking history and chronic alcohol consumption history. Routine haematology assessment included WBC count, haemoglobin, platelet count, bone marrow blast percentage and LDH. Iron/inflammatory markers included serum ferritin, vitamin B12, serum folate, C-reactive protein (CRP) and procalcitonin (PCT).The coagulation panel included prothrombin time (PT), activated partial thromboplastin time (APTT), FIB (unit: mg/dL) and DD. Renal/hepatic function assessment included alanine transaminase, aspartate transaminase, serum albumin, total bilirubin, creatinine (Cr), blood urea nitrogen (BUN) and uric acid. Cardiac biomarkers included B-type natriuretic peptide (BNP) and left ventricular ejection fraction. Immune subsets included absolute CD4+ T cell count, absolute CD8+ T cell count and CD4/CD8 ratio.

**Table 1:** Baseline characteristics of training cohort (n=98).

Variables	Total (n = 98)	Survival group (n = 77)	Death group (n = 21)	Statistic	P
Age, M (Q_1_, Q_3_)	66.50 (49.50, 71.75)	60.00 (45.00, 69.00)	71.00 (68.00, 78.00)	Z=-3.53	<0.001
Treatment stratification, n (%)				χ²=1.86	0.173
Intensive chemotherapy	62 (63.27)	51 (66.23)	11 (52.38)		
Low-intensity HMAs	36 (36.73)	26 (33.77)	10 (47.62)		
Number of Positive Prognostic Genes, M (Q_1_, Q_3_)	2.00 (0.00, 3.00)	2.00 (0.00, 3.00)	0.00 (0.00, 2.00)	Z=-3.10	<0.01
Ferritin, M (Q_1_, Q_3_)	550.08 (311.00, 1029.73)	519.70 (295.37, 871.30)	1005.24 (540.65, 1364.80)	Z=-2.48	0.013
Folate, M (Q_1_, Q_3_)	8.68 (5.32, 18.77)	8.68 (5.39, 19.59)	7.90 (5.09, 15.97)	Z=-0.56	0.574
Vitamin B12, M (Q_1_, Q_3_)	393.74 (174.09, 682.29)	398.00 (171.00, 694.00)	267.32 (183.38, 622.00)	Z=-0.60	0.547
Leukocytes, M (Q_1_, Q_3_)	12.84 (3.28, 51.71)	12.95 (3.34, 51.91)	12.73 (2.80, 42.52)	Z=-0.14	0.886
Haemoglobin, M (Q_1_, Q_3_)	76.50 (61.50, 93.00)	79.00 (60.00, 94.00)	74.00 (68.00, 84.00)	Z=-0.24	0.812
Platelets, M (Q_1_, Q_3_)	45.00 (23.00, 78.75)	48.00 (26.00, 82.00)	42.00 (19.00, 69.00)	Z=-0.92	0.356
LDH, M (Q_1_, Q_3_)	325.50 (202.50, 604.00)	288.00 (175.00, 578.00)	470.00 (246.00, 655.00)	Z=-1.59	0.111
CD4 Count, M (Q_1_, Q_3_)	616.61 (292.45, 1201.83)	619.00 (278.02, 1196.39)	611.94 (315.74, 1205.23)	Z=-0.11	0.910
CD8 Count, M (Q_1_, Q_3_)	414.68 (150.64, 751.32)	408.98 (145.33, 721.44)	420.37 (210.23, 758.80)	Z=-0.11	0.910
CD4/CD8 Ratio, M (Q_1_, Q_3_)	1.71 (1.16, 2.40)	1.56 (1.00, 2.36)	1.81 (1.36, 2.99)	Z=-1.56	0.118
Bone Marrow Blast Percentage, M (Q_1_, Q_3_)	62.75 (43.50, 80.00)	60.00 (43.00, 80.00)	73.00 (48.00, 84.00)	Z=-1.24	0.216
BMI, M (Q_1_, Q_3_)	24.16 (21.73, 26.77)	24.77 (22.14, 27.04)	22.31 (19.61, 25.10)	Z=-2.01	0.044
ALT, M (Q_1_, Q_3_)	18.00 (11.00, 33.25)	18.00 (11.00, 34.00)	18.00 (12.00, 31.00)	Z=0.00	1.000
AST, M (Q_1_, Q_3_)	18.50 (14.00, 35.25)	18.00 (14.00, 33.00)	24.00 (15.00, 36.00)	Z=-0.68	0.496
Albumin, M (Q_1_, Q_3_)	37.15 (33.35, 40.80)	37.50 (33.70, 41.40)	35.70 (32.40, 38.80)	Z=-1.37	0.171
TBIL, M (Q_1_, Q_3_)	13.90 (9.10, 20.45)	14.00 (9.00, 20.50)	13.80 (9.40, 18.70)	Z=-0.07	0.945
Cr, M (Q_1_, Q_3_)	61.00 (50.60, 77.60)	60.40 (49.58, 76.33)	65.70 (53.80, 82.97)	Z=-1.06	0.287
BUN, M (Q_1_, Q_3_)	5.13 (4.21, 6.59)	4.82 (4.05, 6.36)	6.06 (5.27, 7.97)	Z=-2.78	0.005
BNP, M (Q_1_, Q_3_)	528.36 (228.65, 1354.16)	455.00 (131.00, 1129.66)	1150.00 (550.00, 3028.89)	Z=-2.90	0.004
UA, M (Q_1_, Q_3_)	309.00 (219.25, 401.75)	315.00 (210.00, 401.00)	300.00 (249.00, 415.00)	Z=-0.42	0.678
LVEF, M (Q_1_, Q_3_)	63.00 (59.00, 66.00)	63.00 (59.00, 66.00)	65.00 (61.00, 68.00)	Z=-1.41	0.160
PT, M (Q_1_, Q_3_)	12.50 (11.70, 14.30)	12.30 (11.60, 14.00)	13.70 (12.50, 15.40)	Z=-2.40	0.016
APTT, M (Q_1_, Q_3_)	33.10 (29.52, 38.35)	32.20 (29.30, 37.40)	36.80 (34.20, 40.20)	Z=-2.13	0.034
FIB, M (Q_1_, Q_3_)	370.60 (239.08, 455.87)	336.90 (230.60, 443.80)	433.00 (379.20, 477.00)	Z=-2.23	0.026
DD, M (Q_1_, Q_3_)	1.64 (0.60, 7.08)	1.39 (0.55, 5.07)	2.52 (1.09, 16.30)	Z=-1.84	0.066
CRP, M (Q_1_, Q_3_)	29.12 (8.82, 65.86)	22.60 (6.32, 47.58)	92.73 (43.30, 124.91)	Z=-3.90	<0.001
PCT, M (Q_1_, Q_3_)	0.43 (0.18, 4.57)	0.30 (0.15, 3.85)	1.56 (0.56, 10.30)	Z=-2.65	0.008
Genetic Risk Group, n (%)				χ²=10.73	0.005
Favourable	37 (37.76)	35 (45.45)	2 (9.52)		
Intermediate	42 (42.86)	27 (35.06)	15 (71.43)		
Adverse	19 (19.39)	15 (19.48)	4 (19.05)		
Prognostic Gene mNGS Positive, n (%)	65 (66.33)	58 (75.32)	7 (33.33)	χ²=13.03	<0.001
NPM1, n (%)	19 (19.39)	19 (24.68)	0 (0.00)	χ²=4.95	0.026
Extramedullary Disease, n (%)	44 (44.90)	25 (32.47)	19 (90.48)	χ²=22.44	<0.001
Gender, Male, n (%)	56 (57.14)	44 (57.14)	12 (57.14)	χ²=0.00	1.000
Smoking History, n (%)	30 (30.61)	25 (32.47)	5 (23.81)	χ²=0.58	0.445
Drinking History, n (%)	14 (14.29)	9 (11.69)	5 (23.81)	χ²=1.11	0.291
Transplantation, n (%)	2 (2.04)	2 (2.60)	0 (0.00)	–	1.000

M, median; Q_1_, first quartile; Q_3_, third quartile; HMAs, hypomethylating agents; LDH, lactate dehydrogenase; BMI, body mass index; ALT, alanine transaminase; AST, aspartate transaminase; TBIL, total bilirubin; Cr, creatinine; BUN, blood urea nitrogen; BNP, B-type natriuretic peptide; UA, uric acid; LVEF, left ventricular ejection fraction; PT, prothrombin time; APTT, activated partial thromboplastin time; FIB, fibrinogen; DD, D-dimer; CRP, C-reactive protein; PCT, procalcitonin; mNGS, targeted next-generation sequencing; NPM1, nucleophosmin 1.

For the treatment grouping variable, patients were stratified into the intensive induction chemotherapy group and low-intensity hypomethylating agent monotherapy group for subgroup-stratified analysis.

Laboratory testing platforms were uniformly documented, including complete blood counts via a Sysmex XN-9000 haematology analyser, coagulation testing via a Sysmex CS-5100 automated coagulation analyser, biochemical/ferritin/inflammatory markers via a Beckman Coulter AU5800, BNP chemiluminescence via a Beckman Coulter DxI 800 and lymphocyte subset flow cytometry via a Beckman Coulter DxFLEX.

#### 2.3.2 Molecular genetics and definition of number of positive prognostic genes

All patients received a targeted NGS mutation panel and polymerase chain reaction fusion gene detection, covering *FLT3-ITD*, *NPM1*, *CEBPA*, *DNMT3A*, *IDH1/2*, *RUNX1*, *TP53* and other common AML driver mutations.

For the formal definition of positive prognostic genes, consistent with ELN 2017/2022 favourable molecular risk criteria, positive prognostic markers were limited to two mutation categories:

Isolated *NPM1* mutation or *NPM1* mutation with low allele-burden *FLT3-ITD*;

Biallelic double *CEBPA* mutation.

The rationale for excluding *RUNX1::RUNX1T1* and *CBFB::MYH11* core binding factor (CBF) fusion genes was that the institutional NGS mutation panel used in this study only covered somatic gene mutation hotspots and did not routinely perform karyotype or fusion gene fluorescence *in situ* hybridisation (FISH) testing for all patients. Moreover, complete baseline fusion gene data were unavailable for 32% of enrolled patients, introducing missing data bias if incorporated; ELN risk stratification separates CBF chromosomal fusion favourable risk from *NPM1*/*CEBPA* mutation favourable risk, with distinct leukemic proliferation and inflammatory signalling pathways. This study focused specifically on quantifying favourable somatic mutation burden rather than cytogenetic fusion status; hence, fusion genes were excluded from the composite count variable to avoid mixed biological dimensions.

Count scoring rules: each qualifying favourable mutation type counted as 1 point:

0 points = no favourable *NPM1*/*CEBPA* mutations detected,

1 point = single favourable mutation (isolated *NPM1* or biallelic *CEBPA*),

2 points = concurrent *NPM1* + biallelic *CEBPA* mutations,

3 points = *NPM1* mutation + biallelic *CEBPA* + low-burden *FLT3-ITD*.

**Supplementary Table 1** presents the full frequency distribution of positive prognostic gene counts across all 140 patients: 0 points (41 patients), 1 point (66 patients), 2 points (29 patients), 3 points (4 patients).

#### 2.3.3 Standardised extramedullary disease diagnostic criteria

Here, EMD was defined as histopathologically confirmed myeloblast infiltration in anatomical sites outside bone marrow and peripheral blood, including leukaemia cutis, gingival infiltration, central nervous system (CNS) leukaemia, soft tissue, lymph node and solid organ infiltration. All newly diagnosed patients received standardised cross-sectional imaging (contrast-enhanced computed tomography, brain/spine magnetic resonance imaging [MRI] as clinically indicated). All radiologically suspected extramedullary lesions underwent biopsy for histological confirmation; imaging-only suspected lesions without pathological myeloblast verification were not classified as EMD. Diagnostic standards strictly followed WHO myeloid neoplasm classification criteria. Supplementary imaging and pathological diagnostic workflows were documented to reduce ascertainment bias.

### 2.4 Study endpoints

The primary endpoint was 60-day all-cause mortality, defined as death from any aetiology within 60 calendar days of confirmed AML diagnosis. Survival time was calculated from the diagnosis date to the death date or the day 60 follow-up cutoff, whichever occurred earlier.

Secondary endpoints included OS, progression-free survival (PFS) and induction remission status (complete remission (CR)/CR with incomplete count recovery), assessed as per ELN 2022 response criteria. Allogeneic haematopoietic stem cell transplantation during follow-up was recorded as a covariate for subgroup sensitivity analysis.

Remission time was defined as the number of days to achieve post-induction CR and was therefore treated as a post-baseline follow-up outcome rather than a baseline predictive variable. It was not included in univariate Cox screening (**Table 2**).

**Table 2:** Univariate Cox proportional hazards regression analysis.

Variables	β	S.E	Z	P	HR (95%CI)
Extramedullary Disease	2.50	0.74	3.36	<0.001	12.22 (2.84 ~ 52.61)
Age	0.04	0.02	2.33	0.020	1.04 (1.01 ~ 1.08)
Number of Positive Prognostic Genes	-0.41	0.16	-2.47	0.014	0.67 (0.48 ~ 0.92)
Cr	0.00	0.01	0.44	0.659	1.00 (0.99 ~ 1.02)
BUN	0.24	0.10	2.37	0.018	1.27 (1.04 ~ 1.56)
BNP	0.01	0.00	3.01	0.003	1.01 (1.01 ~ 1.01)
PT	0.18	0.09	2.05	0.040	1.19 (1.01 ~ 1.41)
APTT	0.04	0.03	1.33	0.183	1.04 (0.98 ~ 1.10)
FIB	0.01	0.00	2.50	0.012	1.01 (1.01 ~ 1.01)
DD	0.02	0.02	1.33	0.184	1.02 (0.99 ~ 1.06)
CRP	0.01	0.00	2.50	0.013	1.01 (1.01 ~ 1.01)
PCT	-0.00	0.01	-0.20	0.840	1.00 (0.98 ~ 1.01)
Ferritin	0.01	0.00	2.80	0.005	1.01 (1.01 ~ 1.01)
Albumin	-0.04	0.04	-1.00	0.319	0.96 (0.88 ~ 1.04)
BMI	-0.09	0.06	-1.49	0.136	0.91 (0.81 ~ 1.03)
PFS	-1.08	0.51	-2.10	0.036	0.34 (0.13 ~ 0.93)
NPM1	-19.33	7306.48	-0.00	0.998	0.00 (0.00 ~ Inf)
FLT31ITD	-18.27	4754.77	-0.00	0.997	0.00 (0.00 ~ Inf)

β, regression coefficient; S.E, standard error; HR, hazard ratio; CI, confidence interval; Cr, creatinine; BUN, blood urea nitrogen; BNP, B-type natriuretic peptide; PT, prothrombin time; APTT, activated partial thromboplastin time; FIB, fibrinogen; DD, D-dimer; CRP, C-reactive protein; PCT, procalcitonin; BMI, body mass index; PFS, progression-free survival; NPM1, nucleophosmin 1; FLT3-ITD, fms-like tyrosine kinase 3 internal tandem duplication.

### 2.5 Dataset splitting, model construction and overfitting adjustment

The stratified random cohort split was a 7:3 training/hold-out validation split stratified by binary 60-day mortality endpoint via a random number generator, ensuring balanced event proportions between cohorts; the validation set event count (n=8) is clearly reported in the Results section.

For univariate Cox proportional hazards regression, all baseline clinical, laboratory, inflammatory, coagulation and molecular variables with P<0.1 in the intergroup survival comparison were entered to screen candidate predictors.

Multivariate stepwise Cox regression involved forward–backward stepwise variable selection integrating statistical significance and clinical biological plausibility. Collinearity diagnosis (VIF) was performed for all univariate-significant variables; age showed moderate collinearity with ferritin and BNP (VIF>3.5). Following adjustment for EMD, FIB, ferritin and favourable gene count, ‘age’ lost its independent predictive effect and was excluded from the final model. Supplementary stratified age-subgroup analysis (<65 years vs ≥65 years) confirmed the consistent hazard direction and magnitude of the four core predictors across age strata (**Supplementary Table 2**).

To verify the proportional hazards assumption, Schoenfeld residual tests were applied to all variables in the final multivariate Cox model; all variables satisfied the proportional hazards assumption (all P>0.05).

Overfitting correction via multimodal internal validation involved the following:

- Two hundred bootstrap resampling iterations on the training cohort to generate optimism-corrected area under the curve (AUC), C-index and calibration curves;
- Stratified 10-fold cross-validation (stratified by 60-day mortality) to calculate average cross-cohort predictive metrics, mitigating the bias due to the small sample size and limited endpoint events (only 29 total deaths across 140 patients).

Nomogram visualisation was constructed based on adjusted hazard coefficients from the final multivariate Cox model for individualised 60-day mortality risk scoring.

### 2.6 Model performance evaluation

Discrimination included time-dependent 60-day receiver operating characteristic (ROC) curves, raw and bootstrap-corrected AUCs, C-index calculated for training, cross-validation and hold-out validation sets. A direct head-to-head discrimination comparison was made between our nomogram and the ELN 2022 three-tier risk stratification system.

Calibration involved raw and bootstrap-corrected calibration plots comparing model-predicted 60-day mortality probability against observed actual mortality rates.

Clinical utility assessment included decision curve analysis (DCA) curves that plot net clinical benefit across threshold probabilities of 0.05–0.85. A magnified subgraph of the clinically critical low-risk threshold window (0–0.3) was added to visualise the net benefit for routine low/intermediate-risk patients; discussion clarified that the wide upper threshold range (0.3–0.85) corresponds to rare ultra-high-risk patients with EMD-positive multiorgan failure who still gain clinical benefit from intensified supportive intervention.

Interpretability assessment included SHAP analysis using the R ‘shap’ package to calculate global feature importance ranking, individual sample feature contribution and feature–SHAP value dependency scatter plots.

For clinical practicability assessment, a complete real clinical case demonstration integrated nomogram scoring and SHAP individual feature contribution to guide bedside risk assessment and intervention recommendations.

### 2.7 Statistical analysis

All statistical analyses were performed using R software v4.3.3 and Zstats v1.0. Normally distributed continuous variables are presented as mean ± standard deviation, with intergroup comparisons performed using Student’s *t*-test. Non-normal continuous variables are presented as median (interquartile range) (M, Q1, Q3), with intergroup comparisons performed using the Mann–Whitney U test. Categorical variables are presented as *n* (%), with the chi-square (χ²) test or Fisher’s exact test used for small cell counts. Cox regression outputs reported HRs with 95% confidence intervals (CIs) and two-sided P-values. The likelihood ratio test (LRT) was used to evaluate the overall fit of the multivariate Cox model; SHAP values quantified the predictive contribution of features. Two-sided P<0.05 defined statistical significance. Cox proportional hazards regression was used consistently for all time-to-event survival analyses.

## 3 Results

### 3.1 Baseline characteristic comparison of training cohort

The training cohort included 98 patients with AML, stratified into the survival group (*n* = 77) and 60-day death group (*n* = 21). Intergroup comparisons revealed significant differences in multiple core baseline variables.

Regarding demographics, the median age was 71.00 in the death group, compared with 60.00 in the survival group 60.00 (*Z*=−3.53, P<0.001).

Extramedullary disease prevalence was 90.48% (19/21) in the death group versus 32.47% (25/77) in the survival group (*χ²* = 22.44, P<0.001), the most prominent intergroup difference.

Regarding molecular genetics, genetic risk stratification distribution differed significantly (P = 0.005); the median favourable prognostic gene count was 0.00 in the death group versus 2.00 in the survival group (*Z*=−3.10, P = 0.002); the metagenomic NGS favourable gene positivity rate was 33.33% in the death group versus 75.32% in the survival group (P<0.001); NPM1 mutation was exclusively present in the survival group (P = 0.026).

In terms of inflammatory, iron, cardiac and coagulation markers, the death group exhibited significantly elevated ferritin (P = 0.013), CRP (P<0.001), PCT (P = 0.008), BNP (P = 0.004), PT, APTT and FIB (all P<0.05).

In the supplementary stratified treatment subgroup comparison (**Supplementary Table 3**), in both intensive and low-intensity induction subgroups, EMD, elevated FIB/ferritin and low favourable gene count remained significantly associated with 60-day mortality, confirming consistent predictive effects across heterogeneous treatment regimens.

### 3.2 Univariate Cox proportional hazards regression analysis

All variables with P<0.1 in intergroup baseline comparison and clinically recognised AML prognostic markers were incorporated into univariate Cox regression. All univariate regression outputs are presented in **Table 2**.

Extramedullary disease was the strongest univariate risk factor for 60-day mortality (HR = 12.22, 95%CI:2.84–52.61, P<0.001). Age significantly increased ED risk (HR = 1.04 per year, 95%CI:1.01–1.08, P = 0.020). Favourable prognostic gene count was a protective univariate factor (HR = 0.67, 95%CI:0.48–0.92, P = 0.014). Significant laboratory risk markers included BUN, BNP, PT, FIB, CRP and ferritin (all HR = 1.01 per raw-unit measurement); PFS served as a protective univariate factor (HR = 0.34, P = 0.036). Full univariate regression outputs are summarised in **Table 2**.

### 3.3 Multivariate Cox proportional hazards regression analysis

All statistically significant univariate predictors were entered into a forward–backward stepwise multivariate Cox regression, adjusted for collinearity and clinical confounding factors. The final independent predictive variables were EMD, FIB, ferritin and number of positive prognostic genes. Complete multivariate model outputs, including regression coefficient (β), standard error (SE), *Z* statistic, P-value, raw-unit HR, clinically meaningful incremental HR and 95%CI are fully presented in **Table 3**.

**Table 3:** Multivariate Cox proportional hazards regression final model.

Variables	β	S.E	Z	P	Raw HR (95%CI)	Clinically meaningful adjusted HR (95%CI)
Extramedullary Disease	2.11	0.75	2.82	0.005	8.24 (1.91 ~ 35.63)	—
Fibrinogen (per 1mg/dL)	0.01	0.00	3.03	0.002	1.01 (1.01 ~ 1.01)	1.12 (1.04–1.21 per 100 mg/dL increase
Ferritin (per 1ng/mL)	0.01	0.00	2.50	0.012	1.01 (1.01 ~ 1.01)	1.09 (1.02–1.17 per 200 ng/mL increase
Number of Positive Prognostic Genes	-0.52	0.19	-2.74	0.006	0.59 (0.41 ~ 0.86)	—

β, regression coefficient; S.E, standard error; HR, hazard ratio; CI, confidence interval.

Extramedullary lesions were a dominant independent risk factor (β=2.11, SE = 0.75, *Z* = 2.82, P = 0.005, HR = 8.24, 95%CI:1.91–35.63). The wide CI range is attributable to the limited number of total endpoint events (*n* = 29), as detailed in the Discussion section.

Fibrinogen: Raw per 1 mg/dL HR = 1.01 (P = 0.002); clinically meaningful increment per 100 mg/dL rise HR = 1.12 (95%CI:1.04–1.21).

Ferritin: Raw per 1 ng/mL HR = 1.01 (P = 0.012); clinically meaningful increment per 200-ng/mL rise HR = 1.09 (95%CI:1.02–1.17).

Number of positive prognostic genes was an independent protective factor (β=−0.52, SE = 0.19, *Z*=−2.74, P = 0.006, HR = 0.59, 95%CI:0.41–0.86).

In the overall multivariate Cox model, LRT χ²=42.67 and P<0.001, confirming statistically significant global model predictive value. Schoenfeld residual test confirmed all four variables satisfied the proportional hazards assumption (all P>0.05).

### 3.4 Nomogram construction and comprehensive model performance validation

#### 3.4.1 Nomogram

**Figure 2:**
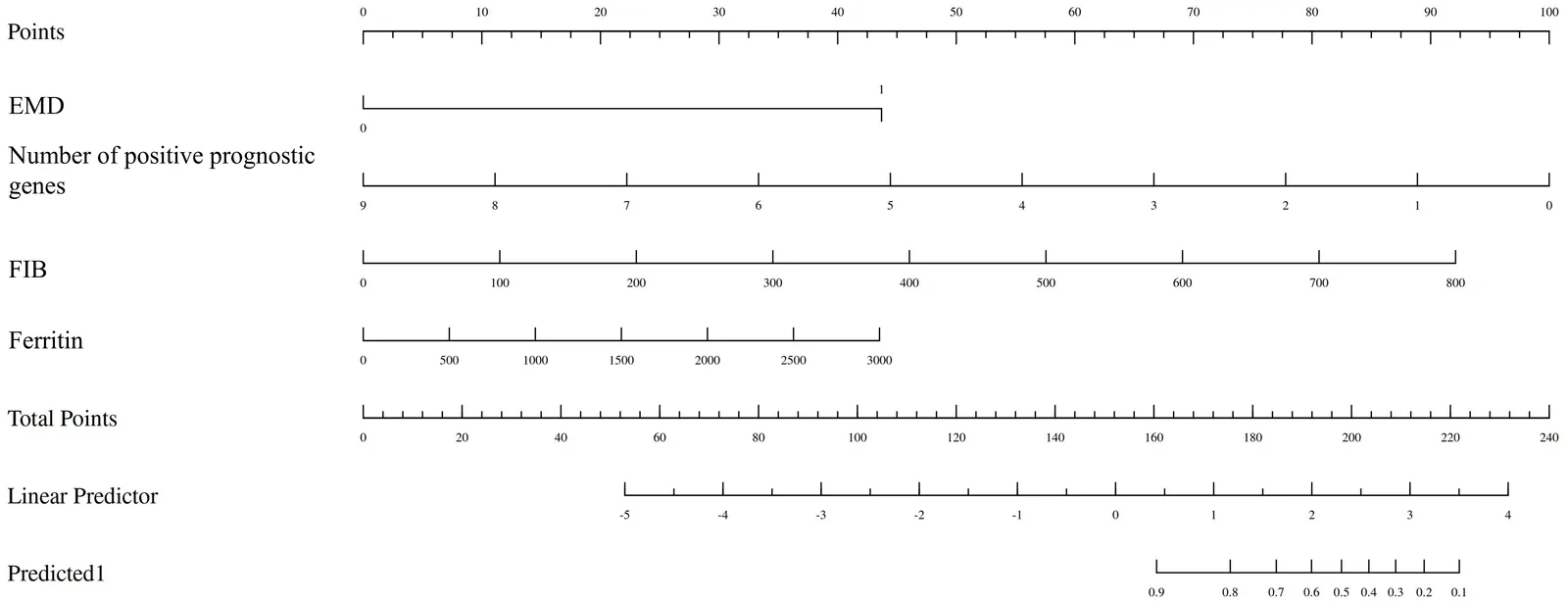
Nomogram for individualised prediction of 60-day all-cause mortality in patients with *de novo* acute myeloid leukaemia.

The visual predictive nomogram is displayed in **Figure 2**.The multivariate Cox regression-derived nomogram was created to visualise individualised 60-day mortality risk scoring. The axis scale for the count of positive prognostic genes covers a range of 0–9, matching the variable distribution shown in **Supplementary Table 1**. The scoring rules were as follows:

Ferritin axis: 0–3,000 ng/mL corresponding score; higher ferritin = higher risk score;

Fibrinogen axis: 0–800 mg/dL corresponding score; higher FIB = higher risk score;

Positive prognostic gene count axis: 0–9; higher count = lower total risk score;

Extramedullary disease: presence = full risk score, absence = 0 score.

The four individual scores were summed to calculate the total points, which were matched to the bottom risk axis to obtain the predicted 60-day mortality probability (0.1–0.9).

To illustrate the clinical application of the nomogram, we present a representative patient case with the following baseline characteristics: The case patient was a 66-year-old patient newly diagnosed with primary AML who was EMD positive (skin leukemic infiltration), with baseline FIB = 430 mg/dL, serum ferritin=980 ng/mL and positive prognostic gene count=1 (isolated *NPM1* mutation without high *FLT3-ITD*).

Step 1 – Nomogram scoring: Ferritin 980 ng/mL=112 points; FIB 430 mg/dL=98 points; gene count of 1 = 21 points reduction; EMD positive=145 points. The total raw score was 334 points, and the corresponding predicted 60-day mortality probability was 0.68.

Step 2 – SHAP interpretation: EMD contributed the largest positive risk increment, elevated FIB and ferritin moderately increased mortality risk and single favourable *NPM1* mutation partially offset risk burden.

Step 3 – Clinical intervention recommendations: enhanced daily organ function monitoring, prophylactic anticoagulation for hyperfibrinogenaemia, iron chelation therapy to reduce ferritin-mediated oxidative stress, reduced-dose induction chemotherapy to minimise treatment-related mortality and weekly cranial MRI screening for CNS leukemic progression.

#### 3.4.2 Model discrimination, calibration and head-to-head European leukaemia network comparison

Regarding training cohort raw performance, AUC = 0.95 (95%CI:0.88–1.02) and C-index=0.86 (95%CI:0.78–0.95). Following 200 bootstrap iterations, the corrected AUC = 0.88 and the corrected C-index=0.79. The stratified 10-fold cross-validation average AUC = 0.87 and the average C-index=0.78.

For the hold-out internal validation cohort (*n* = 42, 8 death events), raw AUC = 0.91 (95%CI:0.78–1.03) and raw C-index=0.82 (95%CI:0.64–1.00); the bootstrap-corrected AUC = 0.84 and the corrected C-index=0.76. Raw and corrected calibration curves closely aligned with the ideal diagonal reference line, demonstrating satisfactory calibration.

Regarding European Leukaemia Network 2022 risk stratification comparative discrimination, the training set ELN-only AUC = 0.74, which was significantly inferior to our nomogram (DeLong test P<0.001), confirming incremental predictive value from integrating EMD, inflammatory/coagulation biomarkers and favourable gene burden beyond conventional cytogenetic/molecular stratification alone.

**Figure 3:**
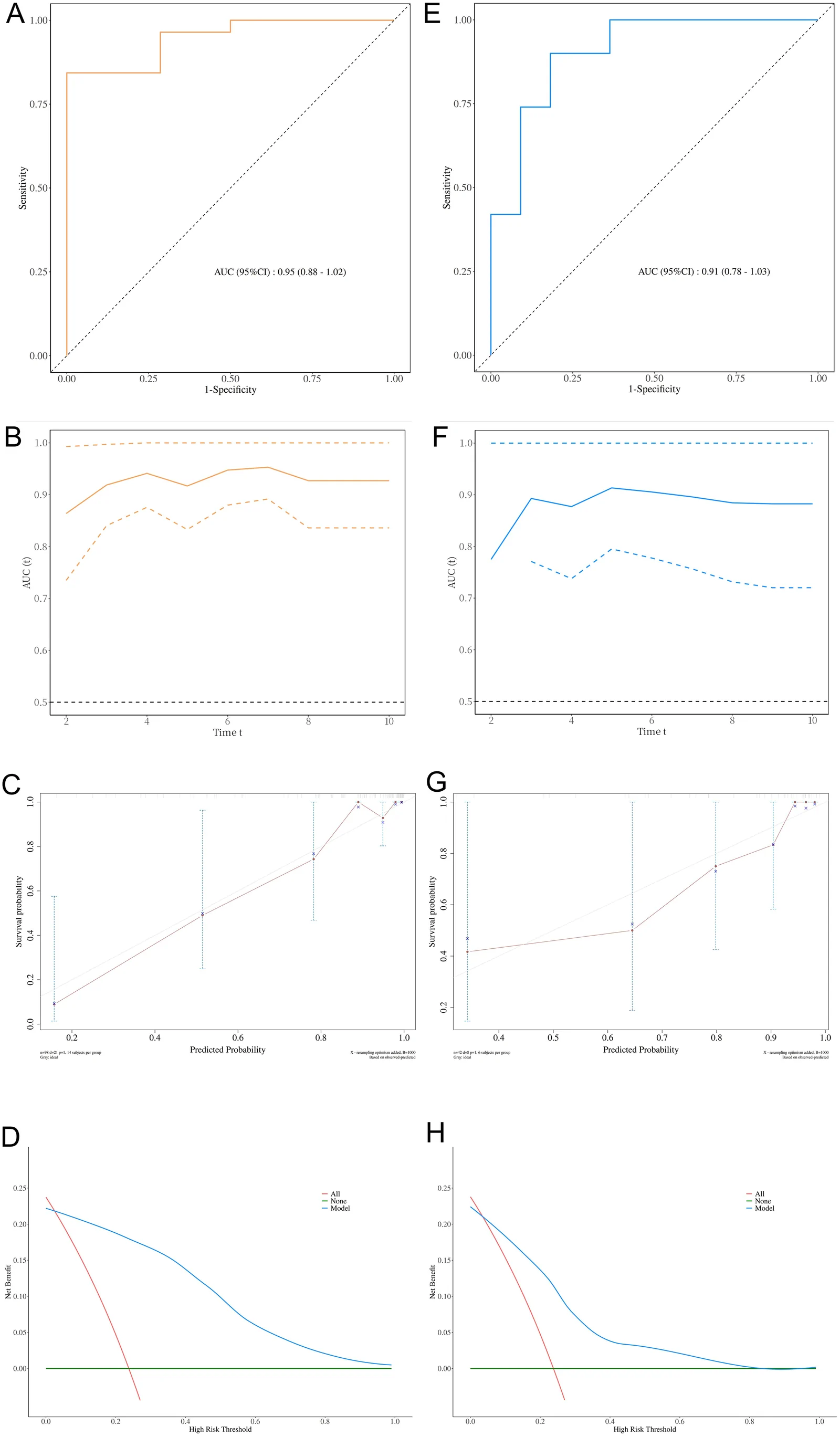
Predictive performance of the model in the training cohort **(A–D)** and internal validation cohort **(E–H)**. **(A, E)** Time-dependent receiver operating characteristic (ROC) curves at the optimal time point, with area under the curve (AUC) and 95% confidence interval indicated. **(B, F)** Time-dependent AUC(t) values across the follow-up period (dashed line: AUC = 0.5 reference). **(C, G)** Calibration plots comparing predicted survival probability with observed survival probability (dashed line: ideal calibration; solid line: bias-corrected calibration). **(D, H)** Decision curve analysis (DCA) showing net benefit across high-risk thresholds; the 0–0.3 threshold range is highlighted to support clinical interpretation.

All ROC, raw and bootstrap-corrected calibration plots for training and validation cohorts are presented in **Figure 3** (subpanels A–G fully labelled with detailed captions)., and complete quantitative performance data are listed in **Supplementary Table 4**.

#### 3.4.3 Decision curve analysis

The DCA curves demonstrated a consistent positive net clinical benefit of the nomogram within the full 0.05–0.85 threshold probability range, superior to universal intervention or no-intervention strategies. The magnified inset subgraph, focusing on the 0–0.3 threshold (routine clinical low/intermediate-risk population), confirmed robust net benefit for the majority of patients with AML. The upper threshold range (0.3–0.85) corresponded to a rare group of patients who were EMD-positive and had multiorgan dysfunction; intensified intensive care unit–level monitoring and supportive care within this high-risk bracket still yielded a net clinical benefit, justifying the broad threshold window observed in this cohort. Additional bootstrap calibration and cross-validation ROC curves are available in **Supplementary Figures 1–S3**.

### 3.5 Shapley additive explanations interpretable machine learning analysis

Shapley Additive Explanations analysis quantified global feature importance and individual predictive contribution for the four nomogram predictors.

**Figure 4:**
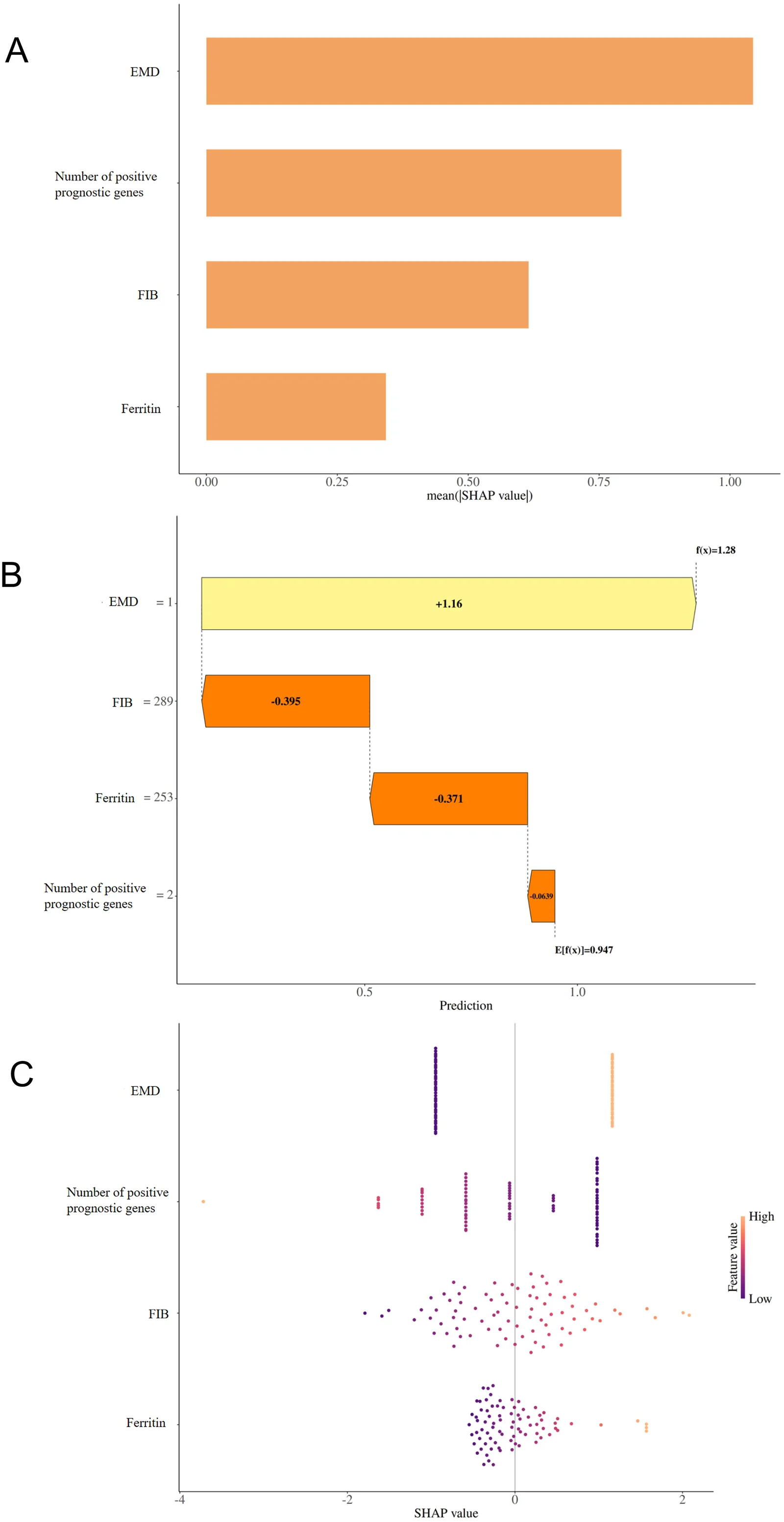
Shapley Additive Explanations (SHAP) interpretable machine learning analysis of the four-predictor nomogram. **(A)** global feature importance ranking; **(B)** individual clinical case waterfall contribution; **(C)** feature–SHAP value distribution.

The global feature importance ranking (**Figure 4A**, average absolute SHAP value) was as follows: extramedullary lesions>number of positive prognostic genes>FIB>ferritin, confirming EMD as the dominant predictive feature driving model risk judgment.

In the individual sample SHAP contribution waterfall plot (**Figure 4B**), yellow bars indicate risk-increasing positive SHAP contributions, and orange bars indicate risk-reducing negative SHAP contributions; EMD positivity generated the largest positive risk increment for individual patients.

In the feature–SHAP dependency scatter plots (**Figure 4C**), binary EMD distribution clearly separated high/low risk SHAP values; FIB and ferritin displayed a positive monotonic correlation with SHAP risk values (higher biomarker levels = higher mortality risk contribution); favourable gene count showed a negative monotonic correlation (higher count = lower predicted risk), consistent with the multivariate Cox regression hazard results.

## 4 Discussion

### 4.1 Core study findings

This single-centre retrospective cohort study constructed and comprehensively internally validated a Cox regression–based nomogram predicting 60-day all-cause early mortality in patients with *de novo* non-APL AML, integrating extramedullary infiltration, FIB, ferritin and favourable prognostic gene count as independent multidimensional predictors. Following bootstrap resampling and stratified 10-fold cross-validation to mitigate overfitting bias due to the limited sample size (*n* = 140) and low endpoint event count (*n* = 29), the nomogram maintained favourable corrected discrimination and calibration across the training and hold-out validation cohorts. A direct head-to-head comparison with the ELN 2022 risk stratification confirmed that our model delivers superior predictive performance by incorporating clinical phenotypic, inflammatory-coagulation and quantitative molecular mutation burden information that is absent from traditional cytogenetic risk frameworks. Decision curve analysis verified broad-spectrum clinical net benefit across all clinically relevant risk thresholds, and SHAP transparent interpretability resolved the ‘black-box’ limitation of conventional prognostic scoring tools (18–21). A complete bedside clinical case demonstration further validated the nomogram’s intuitive, practical clinical utility.

### 4.2 Interpretation of independent predictive variables

Extramedullary disease was identified as the strongest independent adverse predictor (HR = 8.24; highest SHAP global importance). Extramedullary disease reflects intrinsically aggressive leukemic clones with altered tissue homing capacity, high baseline tumour burden and predisposition to multiorgan infiltration and dysfunction, amplifying chemotherapy-related toxicity and ED risk (22–27). In our training cohort, 90.48% of patients who died within 60 days presented EMD at diagnosis, highlighting EMD positivity as an ultra-high-risk clinical phenotype requiring reduced-intensity induction and enhanced multiorgan supportive care, as confirmed by a large multicentre cohort study (Eckardt et al., n=1,583) (10). Consistent with published meta-analyses, EMD independently worsens OS and EFS, with isolated tissue infiltration conferring the worst prognosis. The wide HR 95%CI range (1.91–35.63) is explicitly attributed to the limited total number of 60-day death events (*n* = 29), which reduces the statistical stability of point hazard estimates (see the Limitations section).

Elevated FIB was an independent early mortality risk factor, with a clinically meaningful HR of 1.12 per 100 mg/dL. High FIB reflects systemic AML-driven hyperinflammation and subclinical DIC predisposition, promoting microvascular thrombosis, vital organ hypoperfusion and accelerated ED. Prior meta-analyses have linked FIB to long-term OS impairment; this study first establishes FIB as a bedside-accessible biomarker that specifically predicts ultra-short 60-day mortality, capturing inflammation–coagulation activation and microthrombotic burden at the time of initial diagnosis.

Elevated ferritin independently increased the 60-day mortality risk (per 200 ng/mL, HR = 1.09). Ferritin acts as a dual biomarker of systemic macrophage inflammatory activation and chronic iron overload. Excess free iron generates reactive oxygen species via Fenton chemistry, thereby driving leukemic clonal expansion, immunosuppression, chemotherapy resistance and dysregulation of the ferroptosis pathway in AML blasts. Shapley Additive Explanations scatter plots confirmed a dose–response relationship between ferritin concentration and mortality risk contribution, supporting iron chelation and anti-inflammatory interventions as potential adjuvant supportive strategies for patients with high-ferritin AML (28–32).

Regarding number of positive prognostic genes, quantitative favourable mutation burden served as a robust independent protective factor (HR = 0.59). Unlike conventional ELN categorical risk grouping, this continuous count variable quantifies cumulative favourable molecular signals, demonstrating that multiple concurrent *NPM1*/*CEBPA* mutations exert additive protective effects against early induction-period death, not only long-term OS/EFS. The SHAP analysis ranked favourable gene count as the second most impactful model feature, highlighting the necessity of integrating quantitative molecular burden alongside clinical inflammatory and phenotypic markers for accurate ED risk stratification (33–38).

### 4.3 Multidimensional advantage of the combined nomogram model

The four independent predictors capture three complementary biological dimensions of AML-related early mortality: disease invasive phenotype (EMD), host systemic inflammatory/coagulation/iron metabolic status (FIB, ferritin) and intrinsic leukemic molecular genetics (favourable gene count). No single biomarker or traditional ELN cytogenetic stratification can simultaneously reflect these three domains, explaining the superior discrimination of our nomogram compared with standard risk systems. Rigorous multimodal internal validation (bootstrap correction + stratified cross-validation) mitigated the risk of overfitting due to the small sample size, and stratified subgroup analysis confirmed consistent predictive effects across age groups and induction treatment intensities, demonstrating robust internal stability of the four core predictors. The wide DCA net benefit threshold range (0.05–0.85) confirms the model’s applicability across all risk strata encountered in routine clinical AML practice (39, 40).

### 4.4 Limitations of the present study

The single-centre retrospective design, limited total sample size (*n* = 140) and sparse primary endpoint events (only twenty-nine 60-day deaths) are the main limitations of this study. Furthermore, the study violates the classic Cox regression empirical rule of 10 events per predictive variable, introducing inherent overfitting risk and wide HR CIs (exemplified by the EMD HR CI of 1.91–35.63). Although 200 bootstrap resamples and stratified 10-fold cross-validation were used to generate optimism-corrected performance metrics and reduce overfitting bias, model stability remains constrained by the limited number of events. Multicentre, prospective independent external validation cohorts are required to definitively confirm generalisability, a priority for future research.

In addition, the absence of external multicentre validation limits cross-hospital generalisability. This nomogram is validated only for adult patients with primary *de novo* non-APL AML treated at our single institution. The model cannot be directly extrapolated to secondary AML, APL, paediatric AML or patients managed at geographically distinct medical centres with divergent laboratory testing platforms and induction treatment protocols.

Furthermore, heterogeneous non-standardised induction chemotherapy regimens across enrolled patients represent an important confounding factor for 60-day mortality outcomes. We mitigated this limitation via stratified subgroup analysis separating intensive and low-intensity treatment cohorts, confirming consistent predictor effects across treatment subgroups; however, unmeasured treatment intensity variation may still introduce residual prognostic bias. Future prospective validation studies should implement standardised uniform induction protocols to eliminate treatment heterogeneity.

The custom composite ‘positive prognostic gene count’ variable excludes ELN-favourable CBF fusion genes (*RUNX1::RUNX1T1*, *CBFB::MYH11*) due to incomplete institutional karyotype/FISH data for all patients. This variable only captures somatic *NPM1*/*CEBPA* mutation burden, limiting model applicability to patients with AML primarily stratified via cytogenetic fusion–favourable risk.

Finally, critical standard prognostic covariates (ECOG performance status, full cytogenetic karyotype profiles) were not included in the final multivariate model: baseline ECOG scores had 18% missing data, and complete cytogenetic fusion data were unavailable for a large subset of patients to avoid substantial missing-value bias. Age was excluded after collinearity adjustment and multivariate correction despite univariate significance, with stratified age subgroup analysis provided to partially offset this limitation.

### 4.5 Future research directions

Future research should establish a multicentre prospective external validation cohort with standardised unified inclusion/exclusion criteria, uniform EMD pathological/imaging diagnostic protocols, consistent NGS molecular panels and standardised induction chemotherapy regimens to verify cross-centre generalisability of the nomogram.

Furthermore, the favourable gene composite scoring system should be expanded to integrate CBF chromosomal fusion genes once complete karyotype data are universally available for all patients, refining the quantitative molecular risk metric.

In addition, ECOG performance status and serial changes in inflammatory/ferritin biomarkers during induction therapy should be integrated to construct a dynamic longitudinal ED risk prediction model.

Finally, prospective interventional clinical trials should be conducted to evaluate whether nomogram-guided risk stratification (differentiated supportive care, induction dose adjustment, early prophylactic anti-coagulation/iron chelation) improves 60-day survival in ultra-high-risk patients with EMD-positive AML.

## 5 Conclusion

In summary, this study constructed and multimodally internally validated a novel Cox regression nomogram that integrates EMD status, FIB, ferritin and quantitative favourable prognostic gene count to predict 60-day all-cause early mortality in adult patients with primary *de novo* non-APL AML. Following bootstrap correction and stratified cross-validation to address small-sample overfitting risk, the model exhibited robust discrimination, favourable calibration and broad clinical net benefit superior to conventional ELN 2022 cytogenetic risk stratification. Stratified analysis confirmed stable predictive effects across age and treatment subgroups, and SHAP interpretable machine learning clarified transparent feature contribution mechanisms for clinical practitioners. A complete real clinical case demonstration validated straightforward bedside scoring application. This quantitative, interpretable predictive tool enables early identification of patients with AML at ultra-high risk for ED, supporting individualised adjustment of induction chemotherapy intensity and targeted supportive interventions to improve short-term survival outcomes. Future multicentre prospective external validation is required to confirm broad generalisability across diverse AML patient populations.

## Data Availability

The original contributions presented in the study are included in the article/**Supplementary Material**. Further inquiries can be directed to the corresponding authors.

## Supplementary material

Supplementary Figure 1 Bootstrap calibration curve after 200 resampling iterations.

Supplementary Figure 2 Stratified 10-fold cross-validation average receiver operating characteristic curve.

Supplementary Figure 3 Direct receiver operating characteristic comparison between nomogram and European Leukemia Network 2022 risk stratification.
