# Supplementary material for: Construction and validation of a nomogram model for predicting 60-day mortality in patients with acute myeloid leukaemia
Source: Front Oncol. 2026 Sep 3;16:1846499. doi: 10.3389/fonc.2026.1846499 (PMC13581750; doi:10.3389/fonc.2026.1846499)
Supplement: Supplementary file 1 [file Table1.docx]

**Supplementary Table S1. Frequency distribution of number of positive prognostic genes across the total cohort.**

| **Count of favorable prognostic genes** | **Number of patients** | **Percentage** |
| --- | --- | --- |
| 0 | 41 | 29.29% |
| 1 | 66 | 47.14% |
| 2 | 29 | 20.71% |
| 3 | 4 | 2.86% |
| Total | 140 | 100% |

Note. Favorable prognostic genes include isolated NPM1 mutation, biallelic CEBPA mutation, and NPM1 combined with low-burden FLT3-ITD; Abbreviations: NPM1, nucleophosmin 1; CEBPA, CCAAT/enhancer-binding protein alpha; FLT3-ITD, fms-like tyrosine kinase 3 internal tandem duplication.
