# Supplementary material for: Construction and validation of a nomogram model for predicting 60-day mortality in patients with acute myeloid leukaemia
Source: Front Oncol. 2026 Sep 3;16:1846499. doi: 10.3389/fonc.2026.1846499 (PMC13581750; doi:10.3389/fonc.2026.1846499)
Supplement: Supplementary file 2 [file Table2.docx]

**Supplementary Table S2. Age-stratified subgroup multivariate Cox regression results**

| **Age subgroup** | **Predictor retained** | **Direction of effect** | **Interpretation** |
| --- | --- | --- | --- |
| <60 years | EMD, FIB, ferritin, positive prognostic gene count | Consistent with main model | No reversal of effect direction was observed. |
| >=60 years | EMD, FIB, ferritin, positive prognostic gene count | Consistent with main model | Age stratification supported model stability after age adjustment. |

Note. Abbreviations: EMD, extramedullary disease; FIB, fibrinogen.
