# Supplementary material for: Construction and validation of a nomogram model for predicting 60-day mortality in patients with acute myeloid leukaemia
Source: Front Oncol. 2026 Sep 3;16:1846499. doi: 10.3389/fonc.2026.1846499 (PMC13581750; doi:10.3389/fonc.2026.1846499)
Supplement: Supplementary file 3 [file Table3.docx]

**Supplementary Table S3. Stratified Cox regression by induction treatment intensity**

| **Treatment subgroup** | **Predictor pattern** | **Clinical interpretation** |
| --- | --- | --- |
| Intensive chemotherapy | EMD, high FIB/ferritin, low favorable gene count remained associated with 60-day mortality | Predictive effects persisted under intensive induction. |
| Low-intensity HMAs | Same direction of association across the four predictors | Predictive effects persisted under lower-intensity treatment. |

Note. Abbreviations: EMD, extramedullary disease; FIB, fibrinogen; HMAs, hypomethylating agents.
