# Supplementary material for: Construction and validation of a nomogram model for predicting 60-day mortality in patients with acute myeloid leukaemia
Source: Front Oncol. 2026 Sep 3;16:1846499. doi: 10.3389/fonc.2026.1846499 (PMC13581750; doi:10.3389/fonc.2026.1846499)
Supplement: Supplementary file 4 [file Table4.docx]

**Supplementary Table S4. Validation set event information and optimism-adjusted model performance**

| **Cohort** | **Total patients** | **60-day death events** | **Raw AUC** | **Bootstrap-corrected AUC** | **C-index** | **Corrected C-index** |
| --- | --- | --- | --- | --- | --- | --- |
| Training set | 98 | 21 | 0.95 | 0.88 | 0.86 | 0.79 |
| Internal hold-out validation set | 42 | 8 | 0.91 | 0.84 | 0.82 | 0.76 |
| 10-fold cross-validation average | — | — | — | 0.87 | — | 0.78 |
| ELN 2022 risk only (training) | 98 | 21 | 0.74 | — | 0.69 | — |

Note. Abbreviations: AUC, area under receiver operating characteristic curve; ELN, European Leukemia Network.
